# Impact of FokI (rs2228570) and BglI (rs739837) polymorphisms in *VDR* gene on permanent tooth eruption: A cross-sectional study

**DOI:** 10.1016/j.jobcr.2024.09.012

**Published:** 2024-09-27

**Authors:** Caio Luiz Bitencourt Reis, Kelem Cristina Cambraia Caproni Guerra, Mariane Carolina Faria Barbosa, Fabricio Fernandes Ferreira, Daniela Coelho de Lima, Raquel Assed Bezerra Segato, Ana Cláudia Pedreira de Almeida, Mirian Aiko Nakane Matsumoto, Flares Baratto Filho, Maria Angélica Hueb de Menezes, Erika Calvano Küchler, Daniela Silva Barroso de Oliveira

**Affiliations:** a Department of Pediatric Dentistry, School of Dentistry of Ribeirão Preto, University of São Paulo, Brazil; b Department of Clinic and Surgery, School of Dentistry, Federal University of Alfenas, Brazil; c Department of Pediatric Dentistry, School of Dentistry, Federal University of Minas Gerais, Brazil; d School of Dentistry, Tuiuti University of Paraná, Brazil; e School of Dentistry, Univille University, Brazil; f School of Dentistry, University de Uberaba, Brazil

**Keywords:** Tooth eruption, Single nucleotide polymorphism, vdr

## Abstract

**Introduction:**

Genetic polymorphisms who disturb the mineral homeostasis during tooth development and eruption are candidate to clarify the molecular mechanisms involved in changes in the tooth eruption chronology. In this study, we evaluate whether the FokI (rs2228570) and BglI (rs739837) polymorphisms in the *Vitamin D receptor (VDR)* gene are associated with changes in the chronology of eruption of permanent teeth.

**Material & method:**

This cross-sectional study randomly included 353 biologically unrelated children, both sexes, without systemic impairment or syndromes and history of trauma during the primary dentition. One operator perform the oral clinical examination. The tooth was considered erupted if there was a visible minimum of any tooth surface emerging from the mucosa. Genomic DNA was extracted from buccal epithelial cells from saliva samples. Genotyping was performed by Real-Time Polymerase Chain Reactions using TaqMan® technology. The average of the total number of erupted permanent teeth between the genotypes was compared by the Mann-Whitney test and multivariate Generalized Linear Models (GLM) (α = 5 %). β values with Confidence Interval (CI) 95 % were calculated.

**Results:**

The heterozygous adenine-guanine genotype of the FokI significantly decreases the number of erupted permanent teeth (β = −1.15; CI 95 % = −2.22 to −0.07; p = 0.036). In the stratified analysis for maxillary and mandibular teeth, this genotype was associated with a decrease in the number of erupted maxillary permanent teeth (β = −0.65; CI 95 % = −1.22 to −0.09; p = 0.023). BglI was not associated with permanent teeth eruption.

**Conclusion:**

The FokI, but not BglI, in the *VDR* may delay the eruption of permanent teeth.

## Graphical abstract

**Figure:**
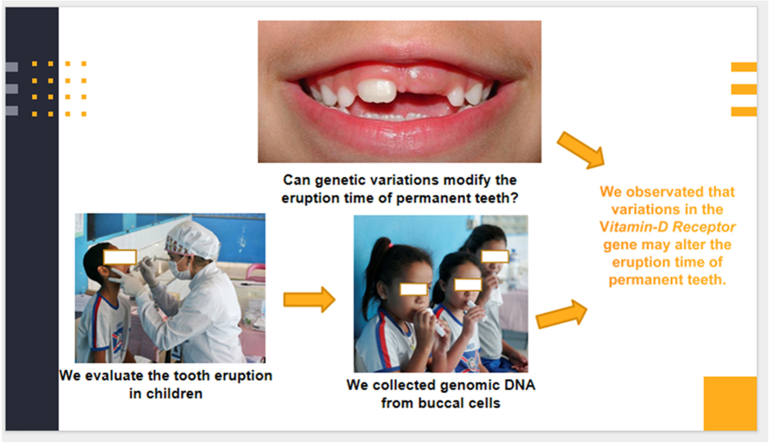

## 1 Introduction

Tooth eruption is a long and complex biological process that involves numerous signaling networks.^1^^,^^2^ It is a finely regulated physiological mechanism that consists mainly of bone resorption until the tooth reaches its final position in the dental arches.^3^^,^^4^ The exact physiological processes of tooth eruption remain inconclusive.^5^ Authors indicate that tooth eruption begins after the start of root formation, which elongates while the tooth moves. In some permanent teeth, an eruption occurs simultaneously with the resorption of the corresponding primary tooth.^6^^,^^7^ This process, under normal conditions, occurs within a defined time interval for each deciduous and permanent tooth. However, some local, systemic, and/or genetic factors can significantly alter the eruption chronology, and may impact the oral health of children and adolescents.^5^^,^^6^^,^^8^, ^9^, ^10^

Vitamin D is a protein that plays a significant role in mineral homeostasis and regulates cell proliferation and differentiation during biological development. Its membrane receptor, the vitamin D membrane receptor (VDR), is present in several cells, and, more specifically, cells involved in tooth eruption, such as periodontal ligament cells,^11^ osteoblasts,^12^ odontoblasts and ameloblasts.^13^ The VDR has already been identified as a participant in the processes of molecular interaction of tooth eruption.^14^ The role of the VDR in tooth eruption is not yet fully understood, however, studies indicate that the receptor acts directly on the cell signaling, molecular transport, and mineral homeostasis during both tooth development and the eruption process.

Polymorphisms in the gene encoding the VDR may modify structurally and functionally the receptor, and decrease, or exacerbate, its affinity for vitamin D, unbalancing all the processes in which the vitamin is involved.^15^ The FokI (rs2228570) and BglI (rs739837) polymorphisms are located in the VDR coding gene and are among the most studied polymorphisms in medicine and dentistry.^10^^,^^11^ FokI and BglI has previously been associated with disturbance of serum vitamin D and calcium levels.^16^ Vitamin D and calcium are exceptional minerals to tooth development and eruption homeostasis.

The investigation of polymorphisms impact on the tooth eruption may clarify the molecular disturbances that alters the chronology of the permanent teeth eruption. Once a polymorphism is identified in a child or adolescent, specific therapies can be applied, and invasive procedures can be avoided, such as orthodontic traction. In this way, our study aimed to evaluate whether the FokI and BglI polymorphisms in the *VDR* gene are associated with changes in the chronology of eruption of permanent teeth in children.

## 2 Methods

This study used the checklist “*STrengthening the REporting of genetic association studies (STREGA): An Extension of the STROBE Statement”* to study design and reporting.

This study was accepted for your execution by the Ethics Committee of the Federal University of Alfenas, Brazil (protocol: 78568217.7.0000.5142). The research was carried out following the Declaration of Helsinki (1964). All children included in this study previously signed the Informed Assent Form, after their parents or guardians had signed the Informed Consent Form.

This is a cross-sectional study that was carried out in the city of Alfenas, located in the southern region of the state Minas Gerais, Brazil. Alfenas has about 79,000 residents with mostly European and African ancestry. The sample was collected in four schools in different regions of the city.

A sample size calculation was previously performed to determine the sample size of the study. The test “Proportions: Difference between two proportions of independent groups” of the G∗Power software version 3.1.9.7 (Franz Faul, University of Kiel, Germany) was applied using the parameters of the allelic distribution of polymorphism between children with and without delay in tooth eruption from the Arid study et al. (2019).^5^ Under an effect size *w* = 0.26, the sample size calculation determined a total n of 334 children needed for this study.

Children aged 8–11 years, both sexes, without systemic impairment or syndromes and biologically unrelated were randomly recruited, regardless of color and race. The random selection process took place by drawing lots of the total number of children enrolled in schools. Due to the dental germ displacement and local disturbances, children who had a history of trauma during the primary dentition were excluded.

Before the oral clinical analysis, a single examiner was trained and calibrated for standardization of diagnoses and reliability of results. The examiner recruited 45 children who were not included in the sample of this study to perform the intra-examiner calibration. The examiner assessed the children twice with an interval of 15 days between assessments and obtained perfect agreement (Cohen's Kappa = 1.0) for analysis of tooth eruption.

The clinical analysis occurred inside the schools under natural light. Gauze, cotton rolls, and a clinical oral mirror were used. Only the eruption of permanent teeth was evaluated. The tooth was considered erupted if there was a visible minimum of any tooth surface emerging from the mucosa, according to Arid. et al. (2019).^5^ Eruption of all permanent teeth was scored as “Yes” or “No”.

Genomic DNA was extracted by buccal epithelial cells from saliva samples following a published protocol.^17^ Briefly, the saliva tube was centrifuged and the supernatant discarded. Proteinase K (100 ng/mL) and an extraction solution (EDTA 5 mmol/L; SDS 0.5 %; Tris-HCl 10 mmol/L, pH 7.8, 1 ml) were added, follow by ammonium acetate for remove the proteins non-digested. The supernatant of this centrifuged tube was separated and the DNA was precipitated with isopropanol. The pellet and the tube were reversed on an absorbent paper to air-dry, and the DNA was resuspended in TE buffer (Tris, 10 mmol/L, pH 7.8; EDTA 1 mmol/L, 100 μL). Spectrophotometry quantified the purity and concentration of the DNA (NanoDrop 1000, ThermoFisher Scientific, Massachusetts, EUA) and stored it at −20 °C.

Genotyping was performed by Real-Time Polymerase Chain Reactions (PCR) (StepOnePlus® Real-time PCR System, Applied Biosystems, Foster City, CA) using TaqMan® technology (Applied Biosystems, Foster City, CA) (TaqMan PCR Master Mix, 2.5 μL; 5 ng DNA in H2O; SNP assay, 0.125 μL). The name of children was replaced by a number for what the operator of this analysis did not recognized the children (blindling method). Two single-base polymorphisms in the *Vitamin D Receptor* (*VDR*) gene were selected according to their potential impact on protein expression and their allelic frequency in the Caucasian population. The FokI (rs2228570) and BglI (rs739837) polymorphisms located in the non-coding region of messenger RNA have previously been associated with low serum vitamin D levels^17^ and high serum calcium levels,^18^ respectively, which can directly interfere with dental development.^10^^,^^11^ Furthermore, these polymorphisms have already been associated with direct alteration of the expression of the VDR and other receptors and proteins in periodontal ligament cells.^11^

**Fig. 1:**
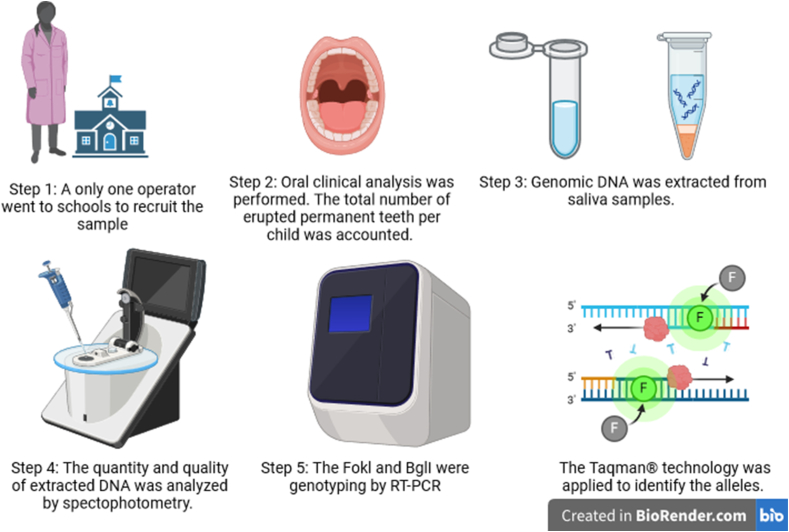
Schematic diagram of study design.

Fig. 1 shows the schematic diagram of study design.

### 2.1 Statistical analysis

Pearson's Chi-square test was applied to assess whether the studied polymorphisms were in Hardy - Weinberg equilibrium.

The erupted permanent teeth of each child were considered a continuous and dependent variable in this study. The mean and standard deviation (SD) of the total number of permanent teeth in the mouth were obtained, which were analyzed using the Shapiro Wilk normality test. After the significant result for normality (p < 0.05), the means were compared between the genotypes by the Mann-Whitney tests. Unadjusted Generalized Linear Models and adjusted for sex and age in months were performed to assess whether genotypes could predict the eruption of permanent teeth in the mouth. The linear model also stratified the results to assess whether there were differences between the upper and lower teeth. The β coefficient, Standard Error (SE), and the 95 % Confidence Interval (95 % CI) were calculated and reported for each linear model. All analyzes were performed using the IBMS SPSS software version 25.0. Values of p < 0.05 were considered statistically significant.

## 3 Results

Five hundred eligible children were recruited for this study. Three hundred and fifty-three children answered the assent form and, their guardians, the consent form. None of them were excluded, therefore, 353 children were included in this study, 170 (48.1 %) boys and 183 (51.9 %) girls. The children's mean age was 8.9 years (SD = 0.89).

The genotyping success rate was 100 % for FokI and 99.4 % for BglI. The two polymorphisms were in Hardy -Weinberg equilibrium.

**Fig. 2:**
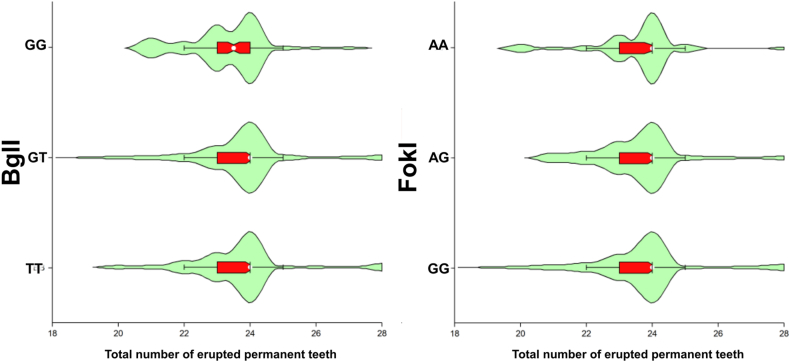
Violins plot to represent the total number of erupted permanent teeth between genotypes.

A comparison of means of total number of erupted permanent teeth between genotypes is shown in Table 1. No statistically significant difference was found between genotypes. Fig. 2 shows violins plot per genotypes.

**Table 1:** Comparison of means of total erupted permanent teeth between genotypes.

Polymorphism	Genotypes	N (%)	Means (SE)	p^#^
FokI	GG	163	15.92 (5.27)	0.129
	AG	144	15.24 (5.37)	
	AA	46	14.50 (5.11)	
BglI	TT	100	15.54 (5.42)	0.598
	TG	169	15.74 (5.52)	
	GG	72	14.56 (4.36)	

Note: # Kruskal-Wallis Test.

The results of the unadjusted and the adjusted generalized linear models by age and sex are shown in Table 2. The adjusted model showed that the heterozygous AG genotype of the FokI polymorphism significantly decreases the number of erupted permanent teeth (β = −1.15; CI 95 % = −2.22 to −0.07; p = 0.036). In the stratified analysis for maxillary and mandibular teeth (Table 3), this genotype was associated only with a decrease in the number of erupted maxillary permanent teeth (β = −0.65; 95 % CI = −1.22 to −0.09; p = 0.023), but not the lower ones (β = −0.49; 95 % CI = −1.05 to −0.06; p = 0.082). BglI was not associated with permanent teeth eruption.

**Table 2:** Unadjusted and the adjusted generalized linear models by age and sex.

Model	Polymorphism	Reference Genotype	Genotypes	SE	β (CI 95 %)	p
Unadjusted	FokI	GG	AG	0.60	−0.65 (−1.84 – 0.52)	0.276
			AA	0.85	−1.03 (−2.70 – 0.63)	0.226
	BglI	TT	TG	0.54	0.25 (−0.90 – 1.42)	0.664
			GG	0.73	−0.76 (−2.02 – 0.67)	0.300
Adjusted	FokI	GG	AG	0.54	−1.15 (−2.22–−0.07)	**0.036**
			AA	0.79	−0.37 (−1.92 – 1.17)	0.634
	BglI	TT	TG	0.53	0.35 (−0.69 – 1.41)	0.506
			GG	0.66	−0.24 (−1.55 – 1.06)	0.716

Note: In bold, p < 0.05.

**Table 3:** – Stratified analysis for maxillary and mandibular teeth.

Model	Polymorphism	Reference Genotype	Genotype	SE	β (CI 95 %)	p
			**Maxillary**			
Unadjusted	FokI	GG	AG	0.31	−0.38 (−1.00 – 0.22)	0.218
			AA	0.44	−0.56 (−1.44 – 0.30)	0.201
	BglI	TT	TG	0.30	0.13 (−0.46 – 0.74)	0.654
			GG	0.38	−0.38 (−1.13 – 0.36)	0.314
Adjusted	FokI	GG	AG	0.28	−0.65 (−1.22–−0.09)	**0.023**
			AA	0.41	−0.23 (−1.05 – 0.58)	0.568
	BglI	TT	TG	0.28	0.20 (−0.35 – 0.76)	0.471
			GG	0.35	−0.12 (−0.81 – 0.56)	0.722
			**Mandibular**			
Unadjusted	FokI	GG	AG	0.31	−0.27 (−0.88 – 0.34)	0.388
			AA	0.44	−0.46 (−1.33 – 0.40)	0.291
	BglI	TT	TG	0.30	0.11 (−0.48 – 0.72)	0.698
			GG	0.38	−0.37 (−1.12 – 0.36)	0.322
Adjusted	FokI	GG	AG	0.28	−0.49 (−1.05 – 0.06)	0.082
			AA	0.41	−0.13 (−0.94 – 0.66)	0.736
	BglI	TT	TG	0.27	0.15 (−0.39 – 0.70)	0.581
			GG	0.34	−0.11 (−0.79 – 0.56)	0.734

Note: In bold, p < 0.05.

## 4 Discussion

The investigation of the relationship between biomolecular factors of tooth eruption, and the causes of imbalances in the eruption chronology is essential to the clarification of the etiology of tooth development disorders.^6^^,^^7^^,^^14^;^19^ Eruption chronology imbalances have direct implications for the clinical practice of pediatric dentists and orthodontists.

The early eruption of permanent teeth can increase the individual's susceptibility to caries, due the most parents or guardians, when faced with the early birth of a permanent tooth, believe that the tooth is a primary tooth, and underestimate proper care and hygiene.^20^ The delayed eruption also has significant implications for the oral health of children and adolescents. When the anterior teeth are affected, there is a psychological problem, by decreasing the child's self-esteem, and social, due to the increased risk of *bullying*. In the field of orthodontics, the tooth delay can affect the space of the dental arches, the verticalization of the antagonist's tooth and delay the treatment of malocclusions.^19^^,^^21^^,^^22^

However, the concept of delay and early tooth eruption should be discussed with caution. Most authors agree with diagnosing a tooth with early or delay of the eruption when it is 2 years ahead or behind the population mean.^6^^,^^7^^,^^14^^,^^19^ Several population averages with tolerance intervals have already been established in Brazil^23^ and reference tables have been generated for dentists as a guide for diagnosis and treatment. However, due to the great miscegenation of the country and the intense secular changes in the growth and development of children and adolescents in the world,^21^ these population averages may not be reliable to be applied in scientific research. Thus, this study aimed to investigate the association of genetic polymorphisms in the *VDR* gene using the total number of erupted permanent teeth as a dependent variable.

The etiology of imbalance of eruption chronology, both in primary and permanent teeth, has been extensively investigated. Several factors have been previously associated such as sex, ethnicity, nutritional status, hormonal deficiencies, local factors^6^^,^^7^^,^^14^^,^^19^ and, more recently, genetic factors.^5^^,^^8^^,^^10^^,^^22^ The most important cells of these processes have the VDR in their membranes, such as osteoblasts, odontoblasts and ameloblasts, osteoclasts, and periodontal ligament cells.^11^, ^12^, ^13^ The VDR is also inserted within an important signaling pathway activated by parathyroid hormone (PTH) during tooth eruption.^14^ In this way, we hypothesized that a failure in the function of the VDR could modify both tooth development and eruption and increase the susceptibility to change the individual's eruption chronology.

The FokI (rs2228570) and BglI (rs739837) polymorphisms are located in the non-coding region of *VDR,* altering the stability and functionality of the mRNA and compromise the coding of protein by ribosomes^24^. FokI has previously been associated with decreased serum vitamin D levels.^17^ It is suggested that this effect is caused due to a hyperfunction of the receptors, which cause negative feedback to the skin cells and decrease their production of vitamin D. The BglI polymorphism has been associated with high serum calcium levels,^18^ demonstrating the impact of the VDR on the PTH signaling pathway, the main calcium-regulating hormone.

This study found statistically significant differences in the number of erupted teeth in children who were heterozygous for the FokI polymorphism compared with homozygous dominant children in the regression analysis adjusted for gender and age. This polymorphism would have the effect of decreasing the eruption potential of permanent teeth, resulting from a decrease in the number of erupted permanent teeth in heterozygous children compared to dominant homozygous children. Polymorphism could exacerbate vitamin D function in cells that would compromise mineral homeostasis of both tooth development and tooth eruption.^17^ Interestingly, when the stratified analysis was performed, an association was only observed in the total number of upper permanent teeth, and not in the lower teeth. Studies indicate that the morphogenesis of each type of tooth is regulated differently by different growth factors and different sets of genes.^25^^,^^26^ Thus, it is reasonable to suggest that polymorphisms may influence the development and eruption of only one type of tooth, or an arch or a group of teeth^27^, ^28^, ^29^ depending on the genotypes or alleles the patient carries.

BglI polymorphism was not associated with tooth eruption in this study, however, we strongly encourage this polymorphism to be studied in different populations due to the potential impact it has on VDR function and, consequently, tooth eruption. Finally, some local conditions such as cysts, odontogenic tumors, tooth agenesis, and supernumerary teeth may be involved in the alteration of the eruption chronology, and are visible only in imaging exams. Although these conditions are uncommon in the population and may not significantly influence the results of our study, the absence of radiographic images can be considered a limitation.

## 5 Conclusion

The FokI (rs2228570), but not BglI (rs739837), polymorphism, of the VDR gene can delay the eruption of permanent teeth, probably due to the impact on the receptor's function to maintain adequate mineral homeostasis during tooth development and eruption.

## Why this paper is important to paediatric dentists and orthodontists

• Eruption chronology imbalances have direct implications for the clinical practice of pediatric dentists and orthodontists.
• The etiology of imbalance of eruption chronology, both in primary and permanent teeth, has been extensively investigated. Several factors have been previously associated such as sex, ethnicity, nutritional status, hormonal deficiencies, local factors, and, more recently, genetic factors.
• The role of the VDR in tooth eruption is not yet fully understood. Studies indicate that the receptor acts directly on the mineral homeostasis of both tooth development and the eruption process. In this way, we hypothesized that a failure in the function of the VDR could modify both tooth development and eruption and increase the susceptibility to change the individual's eruption chronology.

## Ethics approval and consent to participate

This study was accepted for your execution by the Ethics Committee of the Federal University of Alfenas (protocol: 78568217.7.0000.5142). The research was carried out following the Declaration of Helsinki (1964). All children included in this study previously signed the Informed Assent Form, after their parents or guardians had signed the Informed Consent Form.

## Consent for publication

Not applicable.

## Availability of data and material

The datasets used and/or analyzed during the current study are available from the corresponding author on reasonable request.

## Funding

The São Paulo Research Foundation (10.13039/501100001807FAPESP) supported individual scholarship (CLBR, Financial Code: 2021/02704–1).

## Authors' contributions

Conceptualization, E.C.K and D.S.B.O.; methodology, D.C.L., E.C.K., and D.S.B.O. software, C.L.B.R., K.C.C.C.G., F.F.F., A.C.P.A., M.A.N.M., F.B.F., and M.A.H.M.; formal analysis, C.L.B.R. investigation, C.L.B.R., K.C.C.G., anf M.C.F.B.; resources, F.B.F., M.A.H.M., E.C.K and D.S.B.O; data curation, C.L.B.R., M.C.F.B., E.C.K. and D.S.B.O.; writing—original draft preparation, C.L.B.R., K.C.C.G., M.C.F.B., F.F.F.,D.C.L., A.C.P.A., M.A.N.M., F.B.F., M.A.H.M. E.C.K and D.S.B.O; writing—review and editing, C.L.B.R., K.C.C.G., M.C.F.B., F.F.F. ,D.C.L., A.C.P.A., M.A.N.M., F.B.F., M.A.H.M. E.C.K and D.S.B.O; visualization, D.C.L., A.C.P.A., M.A.N.M., F.B.F., M.A.H.M. E.C.K and D.S.B.O.; supervision, D.C.L., E.C.K., and D.S.B.O; project administration, D.C.L., E.C.K., and D.S.B.O; funding acquisition, A.C.P.A., M.A.N.M., F.B.F., D.C.L., E.C.K., and D.S.B.O.

## Declaration of competing interest

The authors declare that they have no known competing financial interests or personal relationships that could have appeared to influence the work reported in this paper.
